# Deciphering the Class III Peroxidase Gene Family and Verifying Their Expression in Modulating Seed Germination in Tomato

**DOI:** 10.3390/antiox14111310

**Published:** 2025-10-30

**Authors:** Jingbo Sun, Feng Zhang, Zhichao Zhao, Mengxia Zhang, Chunjuan Dong

**Affiliations:** State Key Laboratory of Vegetable Biobreeding, Institute of Vegetables and Flowers, Chinese Academy of Agricultural Sciences, Beijing 100081, China

**Keywords:** tomato, class III peroxidases (PRXs), gene family, expression profiling, seed germination

## Abstract

Seed germination is crucial for seedling establishment and is regulated by precise reactive oxygen species (ROS) signaling. Class III peroxidases (PRXs), which are plant-specific enzymes, play crucial roles in plant growth, development, and responses to abiotic stress by maintaining ROS homeostasis. However, members of the PRX gene family in tomato, particularly their functions in modulating seed germination, remain poorly understood. In this study, 102 tomato *PRX*s (*SlPRX*s) were identified, and they were classified into five groups based on phylogenic analysis. Chromosomal localization revealed that these *SlPRX* genes are unevenly distributed across 12 tomato chromosomes, with chromosome 02 harboring the highest densities. Gene structure analysis revealed that *SlPRX*s contain 1 to 10 exons, and *SlPRX4* possesses the most exons. All SlPRX proteins possess the characteristic peroxidase domain and share conserved structural motifs. Collinearity analysis suggested that segmental duplications might be the main contributor to the expansion of the *SlPRX* family. Promoter analysis revealed numerous *cis*-acting elements related to abiotic/biotic stress responses, phytohormones, and growth and development. Notably, seed germination-related elements such as CARE and RY element were identified in some *SlPRX*s. Enzymatic and electrophoresis assays indicated that PRX activity increased with seed germination. Moreover, SHAM, the inhibitor of PRX, exerted an inhibitory effect on tomato seed germination. Transcriptome data revealed stage-specific induction of *SlPRX*s during germination, with distinct expression peaks between 0 and 96 h post imbibition. These findings were further validated by qRT-PCR of the selected *SlPRX* genes. Overall, the findings enhance our understanding of *SlPRX* family members in tomato and highlight their potential for improving seed germination. This study also provides valuable genetic resources and potential molecular markers for breeding tomato varieties with improved germination vigor and stress resilience.

## 1. Introduction

Class III peroxidases (PRXs) are a highly conserved antioxidant enzyme family specifically identified in plants, and they play pivotal roles in plant growth metabolism and stress responses by maintaining homeostasis of reactive oxygen species (ROS) in cells [1]. For instance, PRXs have been reported to participate in a wide range of biological processes, including seed germination [2], root elongation [3], root hair growth [4], fruit ripening [5], lignin biosynthesis [3], and anthocyanin metabolism [6,7]. In addition, PRXs also play crucial functions in defense against diverse abiotic stresses, such as salinity [8,9,10,11], cold [4,9,12,13], heat [11], drought [9,11,14], and pathogen attacks [15]. As the key enzymes in H_2_O_2_ removal, PRXs can use H_2_O_2_ to oxidize a variety of plant secondary metabolites, notably phenolic compounds, and thereby protect plant cells from oxidative damage [16].

The extensive functions of PRXs in the plant life cycle possibly stem from the large number of enzyme isomers in plants (isoenzymes) [1]. For example, 73 members identified in *Arabidopsis*, 119 members in maize [10], 102 members in potato [11], 138 members in rice [16], 75 members in carrots [17], and 374 members in wheat have been identified [14]. Expansion of the *PRX* gene family is mainly dependent on gene replication, through which plants acquire new functions [18], although the redundancy of *PRX* genes often results in a lack of a distinct phenotype associated with the deletion of a single gene in the family.

Seed germination is the beginning stage of the plant life cycle and has a great impact on seedling establishment and the growth status of plants. In agricultural production, the rapid and uniform germination process of seeds is usually regarded as one of the necessary conditions to measure the quality and high yield of crops [19]. It has been well reported that ROS play a vital role in the regulation of seed germination [20]. Low levels of ROS could promote dormancy release and trigger seed germination, while excessive ROS accumulation causes oxidative damages by increasing lipid peroxidation, protein degradation, and DNA and RNA disruption. Therefore, keeping a balance between ROS production and scavenging is necessary to prevent any oxidative damage [21].

Notably, PRXs have been implicated in the regulation of seed germination. In *Arabidopsis*, five class III peroxidases were identified to be involved in early seed germination, of which *AtPRX16*, *AtPRX62*, *AtPRX69*, and *AtPRX71* are repressive for testa rupture and micropylar endosperm rupture, and conversely, *AtPRX4* is responsible for activating these processes [2]. In wheat, a class III *PRX* gene, named *TaPer12-3A*, was identified to be highly expressed in seeds. Overexpression of *TaPer12-3A* significantly improved rice seed germination, while mutations of *TaPer12-3A* promoted dormancy and inhibited germination, implying the positive role of *TaPer12-3A* in seed germination and its negative role in seed dormancy [22]. In rice, Wang et al. reported that overexpression and knockout of *PER1A*, a peroxidase gene, increased seed germinability and decreased seed vigor of transgenic plants [23]. Nevertheless, members of *PRX* genes and their functions in tomato seed germination remain unclear.

Tomato (*Solanum lycopersicum* L.) is an economically important vegetable that is grown worldwide and plays an important role in human nutrition and health by providing vitamins, folic acid, ascorbic acid, and carotenoids. Moreover, the availability of its high-quality reference genome facilitates the comprehensive genomic identification and phylogenetic study of the *PRX* gene family. Furthermore, tomato seed germination is highly sensitive to various abiotic stresses [24], a process where ROS and PRX-mediated signaling are known to play crucial roles [1]. In this study, tomato class III *PRX* (*SlPRX*) gene family members were comprehensively identified using bioinformatics methods, and their physicochemical properties, chromosomal localization, phylogenetic tree, collinearity relationships, and promoter *cis*-acting elements were thoroughly analyzed. In addition, using transcriptomic analysis combined with chemical genetic assay, the expression patterns of *SlPRX*s and their potential functions during seed germination were analyzed. These findings lay the foundation for further exploration of the function of *SlPRX* genes.

## 2. Materials and Methods

### 2.1. Plant Materials, Treatments, and Seed Germination

Tomato seeds ‘Zhongza 9’ were disinfected with 5% NaClO for 10 min, then rinsed with distilled water several times to remove the NaClO on the seed surface. After drying under room temperature, fifty seeds were taken on filter paper and subjected to germination experiments at 28 °C for 10 days. The number of germinating seeds was recorded every 24 h. Germination was signaled by the breakthrough of the radicle through the seed coat.

To evaluate the effect of PRX on tomato seed germination, salicylhydroxamic acid (SHAM), an enzyme-activated inhibitor of PRX [25], was added to Petri dishes at a concentration of 5 μM. Tomato seeds were subjected to germination experiments, and then the germinating seeds at different time points (8, 24, 48, 72, and 96 h) were drained of surface water and quickly frozen in liquid nitrogen for subsequent experiments. The experiments were performed in three biological replicates, with 50 seeds contained in each replicate.

### 2.2. Candidate Gene Screening and Chromosomal Localization

AtPRX protein sequences were downloaded from the TAIR-home database (https://www.arabidopsis.org/ accessed on 17 October 2024) and submitted to phytozome13 (https://phytozome-next.jgi.doe.gov/ accessed on 17 October 2024) using the BLAST tool in phytozome 13 (https://phytozome-next.jgi.doe.gov/blast-search accessed on 17 October 2024), with comparisons performed with the ITAG4.0 database. The theoretical isoelectric point (p*I*) and molecular weight (Mw) of tomato PRX proteins were calculated using the Expasy-Compute website (https://web.expasy.org/compute_pi/ accessed on 19 October 2024) [26]. Subcellular localization of tomato PRX proteins was predicted using the WoLF PSORT site (https://wolfpsort.hgc.jp/ accessed on 19 October 2024) [27].

Gene length, CDS size, protein length, and the chromosomal locations of *SlPRX* were obtained from the ITAG4.0 database. Tomato gene density information was counted using the Gene Density Profile tool in TBtools-II, and the location of *SlPRX* genes on the chromosome was visualized using the Gene Location Visualize tool [28].

### 2.3. Phylogenetic Tree Construction and Gene Structure Visualization

Comparison of tomato and *Arabidopsis* protein sequences was performed with MEGA software (version 11.0.13), and the Neighbor-Joining phylogenetic tree was constructed by using the bootstrap method, setting the number of tests to 1000. The obtained phylogenetic tree was uploaded to the evolview website (https://www.evolgenius.info/evolview/#/treeview accessed on 12 November 2024) to optimize the phylogenetic tree [29,30,31].

The protein sequences were uploaded to MEME Suite 5.5.7 (https://memesuite.org/meme/tools/meme accessed on 8 November 2024), and the number of motifs was set to 10 to obtain motif-related information [32]. The Batch CD search tool of NCBI (https://www.ncbi.nlm.nih.gov/Structure/bwrpsb/bwrpsb.cgi accessed on 8 November 2024) was used to analyze the conserved motifs of genes. The motifs and conserved domains were then visualized using Gene Structure View (Advanced) in TBtools-II [33].

### 2.4. Synteny Analysis

For the interchromosomal relationships of tomato *SlPRXs*, synthetic analysis was conducted by using TBtools-II, and a syntenic map with gene density information was obtained from Gene Density Profile and the Mutiple Synteny Plot tool in TBtools-II.

For the interspecific collinearity analysis, the genome sequences, genome annotation information, and PRX coding sequences for potato were downloaded from SpudDB (https://spuddb.uga.edu/download.shtml accessed on 17 October 2024), and those for *Arabidopsis*, rice, and maize were downloaded from EnsemblPlants (http://plants.ensembl.org/index.html accessed on 17 October 2024). Genome assemblies of all the above species were utilized to calculate synteny and collinearity and to draw a dual synteny plot using the Mutiple Synteny Plot of TBtools-II.

### 2.5. Promoter Cis-Element Analysis

The 2000 bp upstream nucleotide sequences of *SlPRX* genes were extracted from the tomato genome database. Then, they were uploaded to the Plant CARE website (https://bioinformatics.psb.ugent.be/webtools/plantcare/html/ accessed on 8 November 2024) [34,35], and the promoter *cis*-elements of the genes were analyzed using the Search for CARE function to decipher and organize the obtained files, followed by visualizing the *cis*-elements using R studio for statistics and heatmaps.

### 2.6. RNA-Seq

Total RNA of tomato seed samples was prepared using an RNA prep Pure Polysaccharide Polyphenol Plant Total RNA Extraction Kit (Tiangen, Beijing, China, Catalog No. DP441). RNA quality was assessed using the Agilent^®^ 2100 bioanalyzer (Agilent Technologies, Santa Clara, CA, USA). Sequencing libraries were generated using the NEBNext^®^ Ultra™ RNA Library Prep Kit for Illumina^®^ (NEB, Ipswich, MA, USA), and the individual barcoding code sequences were added to each sample. The mRNA was isolated using poly-T magnetic beads, followed by fragmentation and cDNA synthesis. After end repair, adenylation, and adapter ligation, library fragments of ~370–420 bp were selected with AMPure XP system (Beckman Coulter, Beverly, CA, USA). Then, PCR was performed with Phusion™ High-Fidelity DNA Polymerase, Universal PCR primers and Index (X) Primer. Finally, the PCR products were purified using AMPure XP system, and library quality was rechecked before clustering on a cBot Cluster Generation System using TruSeq PE Cluster Kit v3-cBot-HS (Illumia Inc., San Diego, CA, USA).

Sequencing was conducted on the Illumina Novaseq 6000 platform to generate 150 bp paired-end reads. Raw reads were processed with fastp to remove adapters and low-quality sequences. Clean reads were aligned to the tomato genome (GCA_000188115.3) using Hisat2 v2.0.5. Gene-level read counts were obtained via Fea-tureCounts v1.5.0-p3, and fragments per kilobase of transcript sequence per millions base pairs sequenced (FPKM) values were calculated. Finally, *SlPRX* gene expression data were normalized and visualized as a heatmap using R.

### 2.7. qRT-PCR Assay

To verify the accuracy of the transcriptome, 12 *SlPRX* genes related to tomato seed germination were selected for RT-qPCR experiments. The RT-qPCR analysis was conducted on a LightCycler^®^ 96 instrument (Roche Diagnostics, Basel, Switzerland), with a PerfectStart^®^ Green qPCR SuperMix Kit (TransGen Biotech, Beijing, China, Catalog No. AQ601). The primer sequences were designed using the QuantPrime online tool and are listed in Supplementary Table S1. *Actin-41* gene (*Solyc04g011500*) was used as an internal reference, and the relative expression level was calculated using the 2^−∆∆Ct^ method. Three biological replicates were performed per gene, and three technical replicates were performed within an experiment.

### 2.8. PRX Activity Determination and Isozyme Electrophoresis

PRX extraction and determination were performed according to a previous report [36], with some modification. Seed samples (0.1 g) were ground in liquid nitrogen and extracted with 0.5 mL phos-phate buffer (0.1 M, pH 6.0). The supernatant was used to measure peroxidase activity by the guaiacol colorimetric method, using a Plant Peroxidase Assay Kit (Yuanye, Shanghai, China, Cat. No. R30311) according to the manufacturer’s instructions. A boiled extract (100 °C, 10 min) served as the negative control.

Peroxidase isozymes were separated by native PAGE and stained in 0.1 M sodium acetate buffer (pH 5.5) containing 3 mM DAB and 0.03% H_2_O_2_ for 20 min [37]. Gels were visualized under visible light using a Universal Hood II transilluminator (Bio-Rad, Hercules, CA, USA).

### 2.9. Statistical Analysis

All data were presented as the mean  ±  SD. Statistical analysis was performed using SPSS (version 26.0). Data were analyzed using one-way analysis of variance (ANOVA), and Student’s *t*-test and Duncan’s test were used for comparisons. *p*  <  0.05 was considered statistically significant.

## 3. Results

### 3.1. Genome-Wide Analysis Identified 102 SlPRX Genes in Tomato

Using 73 AtPRX protein sequences as queries, 102 *SlPRX* genes were identified in the tomato genome ITAG4.0, named as *SlPRX1* to *SlPRX102* according to their physical locations on the chromosomes (Supplementary Table S2).

**Figure 1:**
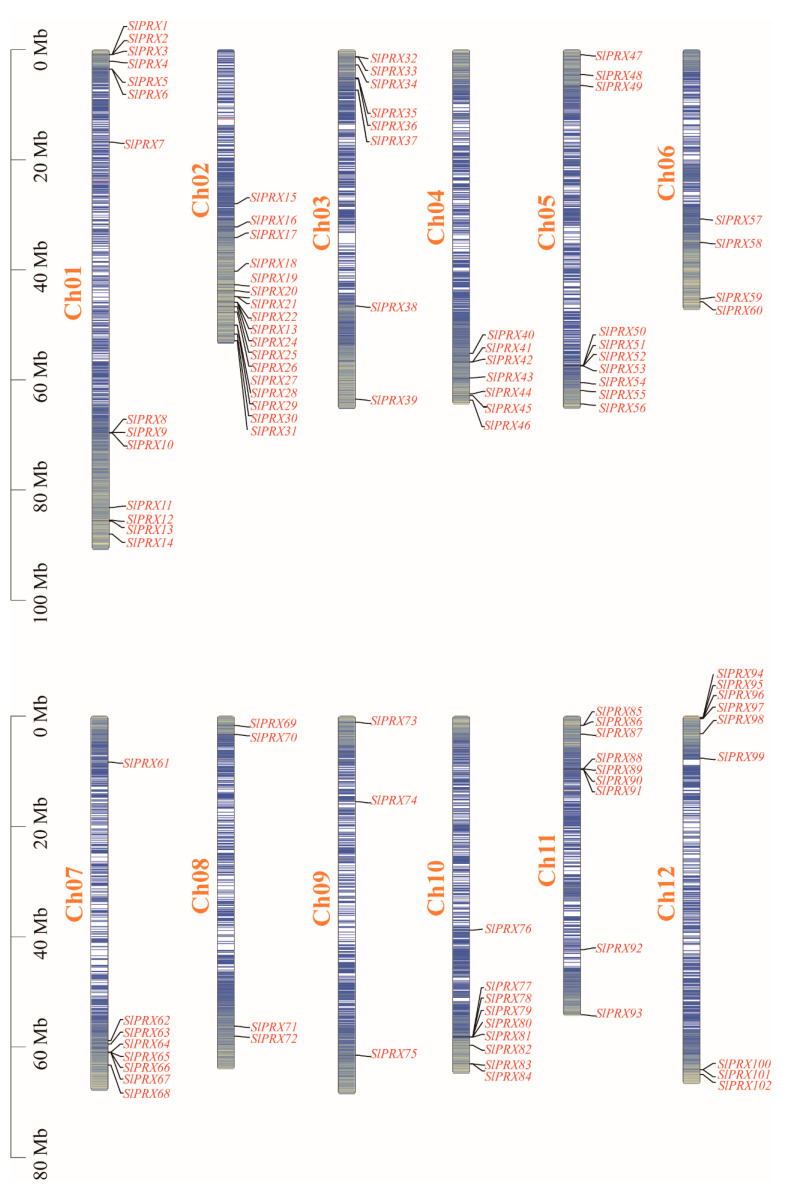
Chromosome localization of *SlPRX* genes. The chromosome numbers are indicated on each vertical bar. The scale of the chromosomes on the left is in megabases (Mb). The genes are unevenly distributed across the genome, with *SlPRX* clusters observed on several chromosomes.

The 102 *SlPRX* genes were located on twelve tomato chromosomes (Figure 1). Chromosome Ch02 contained the maximum number (17) of *SlPRX* genes, and Ch09 contained the minimum number (3). A total of 34 *SlPRX* genes were distributed in the 0–20 Mb region of 12 chromosomes, and 58 genes were distributed in the 40–80 Mb region. Only four *SlPRX* genes (*SlPRX11* to *SlPRX14*) were located at the lower end, within a region between 80 and 100 Mb of chromosome Ch01, longer than the other chromosomes, and six *SlPRX* genes (*SlPRX15* to *SlPRX18*, *SlPRX 57*, and *SlPRX58*) were distributed within a region between 20 and 40 Mb (Figure 1).

The *SlPRX* genes ranged in length from 960 bp (*SlPRX69*) to 10,122 bp (*SlPRX39*), with the vast majority of genes in the 1000–4000 bp range. *SlPRX7* encoded the longest protein (956 aa), which showed the largest MW of 108.77 kDa, while *SlPRX76* encoded the smallest protein with an MW of 26.26 kDa. The isoelectric point (p*I*) of these proteins ranged from 4.52 to 9.87, with the lowest p*I* found in SlPRX1 and the highest p*I* in SlPRX79 (Supplementary Table S3). Subcellular localization prediction indicated that 38 SlPRXs were located extracellularly, while 34 SlPRXs were in chloroplasts. Only SlPRX43 and SlPRX59 were localized in the endoplasmic reticulum, and only one SlPRX was in the peroxisome (SlPRX69) and nucleus (SlPRX95) according to the prediction results (Supplementary Table S2).

### 3.2. Phylogenetic Classification of SlPRXs Highlights Evolutionary Conservation and Divergence

**Figure 2:**
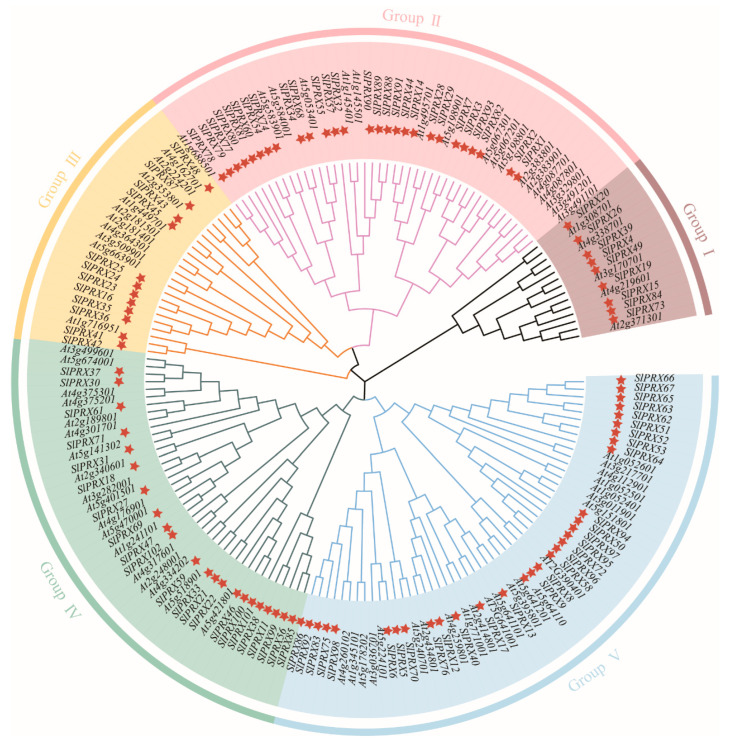
Phylogenetic analysis of class III peroxidase proteins from tomato and Arabidopsis. An unrooted phylogenetic tree was constructed using the neighbor-joining method in MEGA 11, based on the full-length amino acid sequences of PRX proteins from tomato (Sl) and Arabidopsis (At). Bootstrap values from 1000 replicates are shown at the branches to indicate confidence levels. The red stars represent members of the SlPRX gene family from tomato.

To further analyze the evolutionary relationship of the *SlPRX* gene family, the protein sequences of 102 SlPRXs from tomato and 73 AtPRXs from *Arabidopsis* were used to construct an NJ phylogenetic tree. As shown in Figure 2, the corresponding tree divided these PRX proteins into five groups (I-V). Of these groups, group V was the largest one, including 31 SlPRXs and 22 AtPRXs, while group I was the smallest one, containing 9 SlPRXs and 5 AtPRXs. For groups II, III, and IV, 44, 23, and 41 PRXs were included, respectively.

### 3.3. Gene Structure and Conserved Motif Analysis of SlPRX Genes

**Figure 3:**
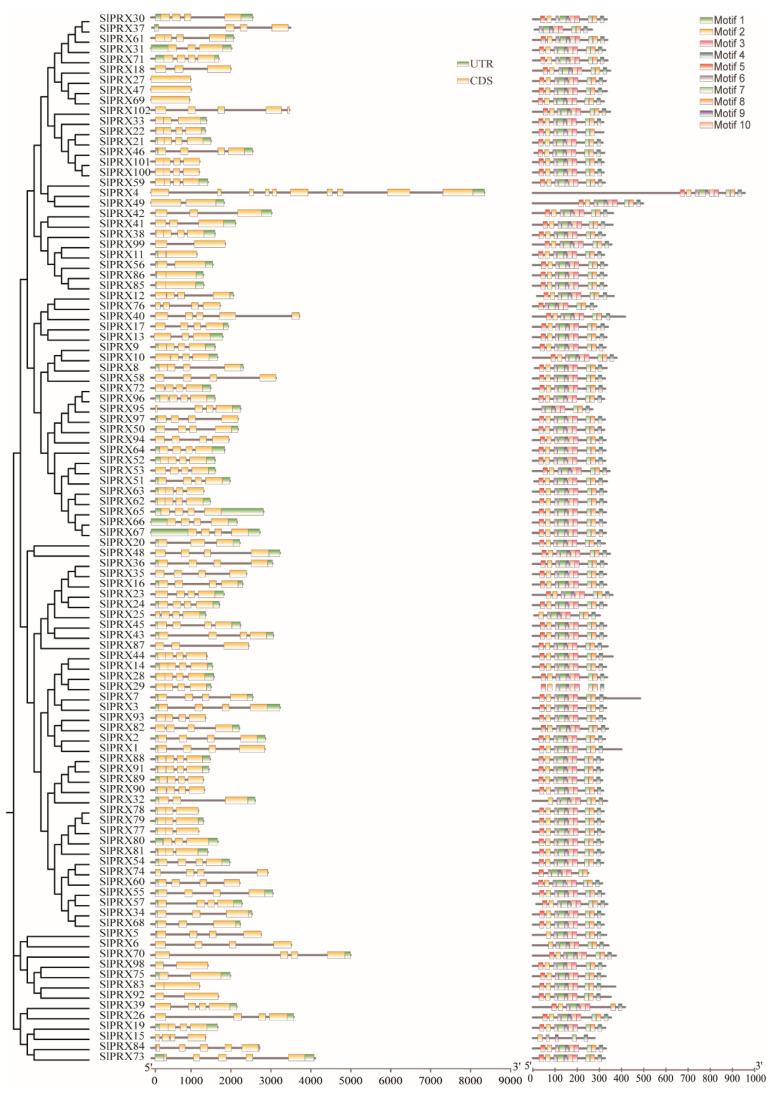
The gene structures and conserved motifs of *SlPRX* genes. The phylogenetic tree in the left was constructed based on the full-length sequences of SlPRX proteins. The exon–intron structure of the SlPRX genes and the conserved motifs of SlPRX proteins were visualized using TBtools software (version 2.310). Untranslated regions (UTRs), coding sequences (CDS), and introns are represented by green boxes, yellow boxes, and black lines, respectively. Distribution of the ten conserved motifs (1–10) was identified by using the MEME database, with each motif represented by a colored box.

A separate phylogenetic tree was constructed using the protein sequences of all the identified *SlPRX* genes, and their exon–intron structures were compared. As shown in Figure 3, *SlPRX* genes possess a variable number of 1 to 10 exons. *SlPRX4* contains the most exons, while for the *SlPRX27*/*SlPRX47*/*SlPRX69* branch, only one exon is present. Of the 102 *SlPRX* genes, more than half (67) of the *SlPRX* genes contain four exons.

Furthermore, we analyzed the conserved motifs of SlPRX proteins using the MEME program (Figure 3). Most of the SlPRX proteins contain 8–10 motifs, with SlPRX 15 having only 5 motifs. Motifs 1, 3, 8, and 10 are present in all 102 SlPRX proteins. Most paralogous proteins contain the same motifs, with the exception of SlPRX15, which contains only motifs 1, 3, 8, and 10. Motifs 1, 2, 5, 7, and 9 are most commonly observed in the N-terminus, and motifs 4, 7, and 8 are present in the C-terminus. Motif 1 and motif 4 can respond to high temperature and oxidative stresses, and motif 9 is reported to bind to antioxidant-responsive elements in response to H_2_O_2_ and oxidative stress.

### 3.4. Collinearity Analysis Unveils Evolution and Expansion History of SlPRX Genes

**Figure 4:**
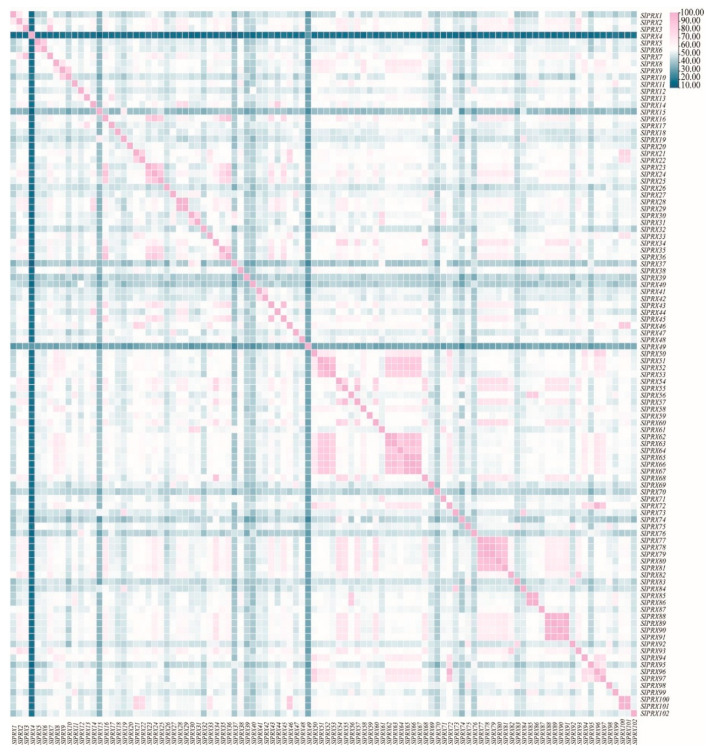
Sequence similarity and correlation between SlPRX proteins. A hierarchical cluster analysis was performed based on the pairwise correlation coefficients of SlPRX protein sequences. The heatmap color scale from blue (high correlation) to pink (low correlation) illustrates the degree of sequence similarity between the different SlPRX proteins.

SlPRX proteins exhibited 13.3–100% identities (Figure 4). Among them, SlPRX88 and SlPRX91 possess the same protein sequence, with a sequence identity of 100%. Three pairs of genes, SlPRX65/SlPRX66, SlPRX78/SlPRX79, and SlPRX85/SlPRX86, showed high sequence similarity, more than 99%. Most of the genes with high sequence similarity are in close proximity to each other on the chromosome (Figure 1). The lowest sequence identities were found between SlPRX4 and SlPRX37 and between SlPRX4 and SlPRX74.

**Figure 5:**
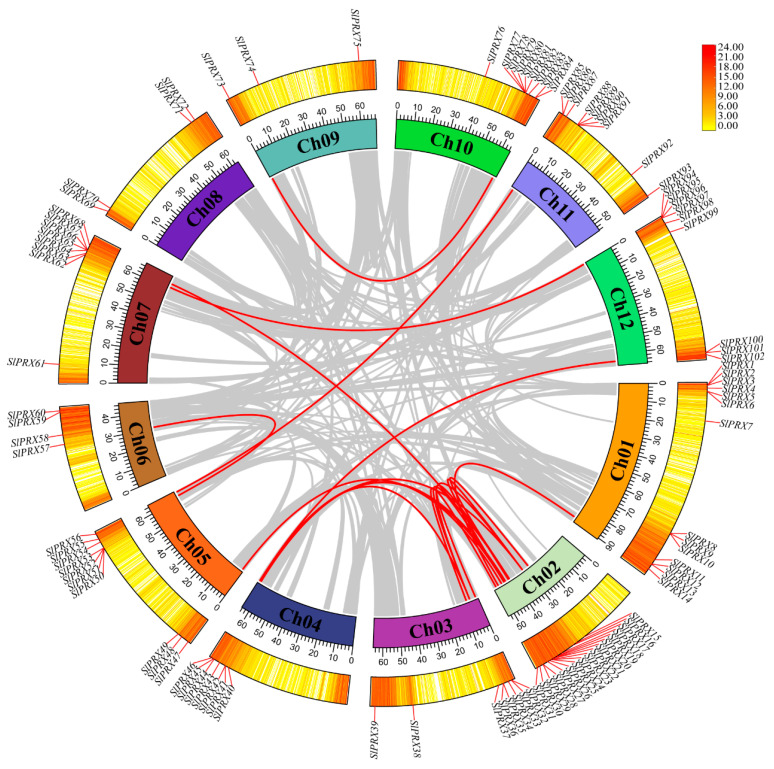
The interchromosomal collinearity relationships of *SlPRX* genes. The syntenic map was visualized using TBtools-II. The outer circle indicates gene density, which is represented by a yellow-to-red color scheme, with a redder color denoting a higher level of gene density. Gray lines connect syntenic genomic regions, and red lines highlight collinear *SlPRX* gene pairs.

To further understand the evolutionary relationships of the *SlPRX* gene family, collinearity analysis was performed within and between species. As shown in Figure 5, the internal collinearity analysis revealed 18 pairs of fragment copy genes among the 102 *SlPRX* gene family members. Among the 12 chromosomes, chr02 had the most fragment replication genes, with 11 pairs. Chr09, chr10, and chr11 each had one pair of fragment replication genes. There was no *SlPRX* fragment replication gene in chr08 (Figure 5).

**Figure 6:**
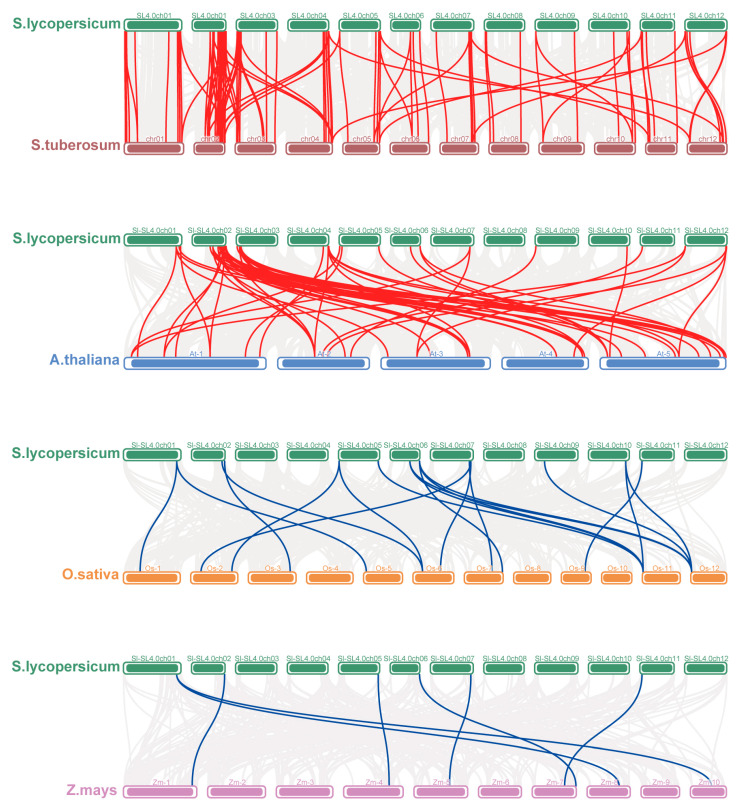
The interspecific collinearity relationships of *PRX* genes. The syntenic relationships of *PRX* genes with tomato and four other plant species (potato, Arabidopsis, rice, and maize), visualized by using TBtools-II. Gray lines represent background syntenic blocks. Red lines connect collinear *PRX* gene pairs with tomato and other dicots (potato, Arabidopsis), and blue lines connect tomato with monocots (rice, maize).

Further collinearity analysis between tomato and other plants was performed (Figure 6). Two dicotyledonous plants (potato and Arabidopsis) and two monocots (rice and maize) were included. Specifically, 102 tomato *SlPRX* genes formed 99 collinear gene pairs with potato, another Solanaceae plant. Of these collinear gene pairs, 72 pairs were on the same chromosomes with a similar gene locus, suggesting that the *PRX* genes of the two species are likely to have evolved from the same ancient *PRX* gene. When compared with Arabidopsis, 52 gene pairs were found, of which *SlPRX16*, *SlPRX23*, and *SlPRX35* genes formed four gene pairs with the corresponding *AtPRXs.* For the two monocotyledons, tomato formed only 19 and 7 collinear gene pairs with rice and maize, respectively. *SlPRX30*, *SlPRX55*, and *SlPRX87* formed collinear gene pairs with four other species, suggesting that the replication events of these three genes occurred before those of the other genes.

### 3.5. Promoter Cis-Element Profiling Implicates SlPRX Genes in Stress Response, Hormone Signaling, and Development

**Figure 7:**
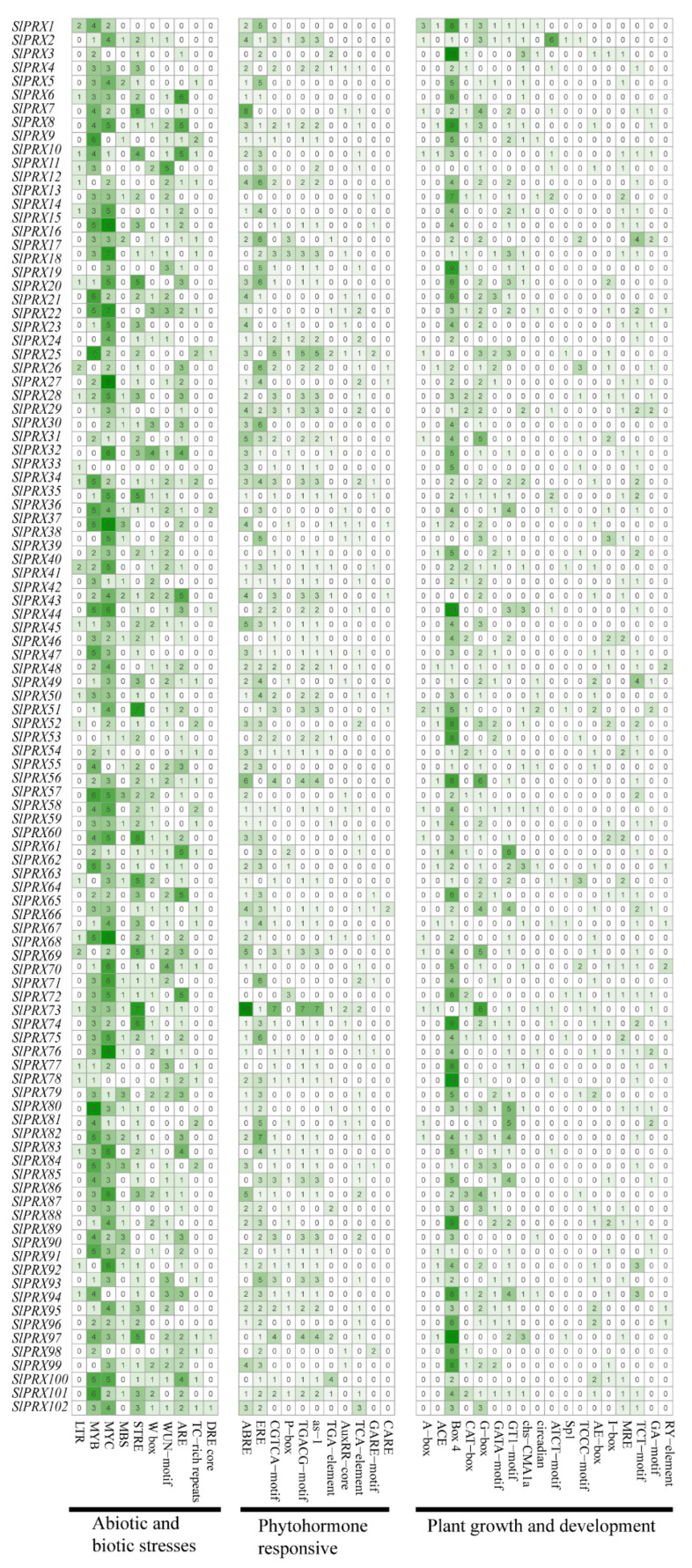
Putative regulatory cis-elements in the promoters of *SlPRX* genes. Blocks with different colors and quantities represent the number of different elements contained within the 2000 bp upstream region of each *SlPRX*. The detailed information for each *cis*-element is available in Supplementary Table S3. The intensity of green color represents the number of cis-elements, with darker shades denoting higher values.

A total of 39 *cis*-elements were found by analyzing the 2000 bp upstream of 102 *SlPRX* genes. As shown in Figure 7, these *cis*-elements were classified into three categories: responsive to abiotic and biotic stresses, phytohormone-responsive, and plant growth and development (Supplementary Table S3; Figure S2).

Abiotic and biotic stress-responsive elements contain 10 *cis*-elements (Figure 7). A total of 88 *SlPRX* genes contain MYB and 94 *SlPRX* genes contain- MYC binding sites. Only 27 *SlPRX* genes possess low-temperature-responsive element (LTR), and 28 genes have TC-rich repeats which are involved in defense and stress responsiveness [38]. *Cis*-element responsive to drought and high-salinity stress (DRE) [9] was found in the promoter of *SlPRX25*, *SlPRX37*, *SlPRX44 SlPRX97*, and *SlPRX102*. Of the 102 *SlPRX* genes, *SlPRX22* possesses the most *cis*-elements related to abiotic and biotic stress responsiveness, while *SlPRX33* has only one.

Phytohormone-responsive elements contain 11 *cis*-elements. A total of 76 *SlPRX* gene promoters possess abscisic acid-responsive element (ABRE), 13 *SlPRX* promoters have gibberellin-responsive element (GARE), and 7 *SlPRX* gene promoters have the CAACTC regulatory element (CARE), which has been reported to be a regulatory element for GA-induced hydrolase gene expression in germinating seeds [39]. A total of 43 hormone-responsive elements were identified in the promoter of *SlPRX73*, including 16 ABREs. The *SlPRX7* promoter contains eight ABREs, while *SlPRX14* has only one GARE in its promoter (Figure 7).

A total of 18 elements involved in plant growth and development were also identified in *SlPRX* gene promoters (Figure 7). Among these *cis*-elements, 96 *SlPRX* promoters possess box4 and 71 *SlPRX* promoters possess G-box, which are both involved in light responsiveness [40]. Nine *SlPRX* promoters contain RY elements, which have been reported to regulate gene expression during late embryogenesis and seed development via ABI3 and FUS3 transcription factors [41]. CARE is involved in seed-specific gene expression, and was found exclusively in the promoter regions of hydrolase genes that were expressed in germinating seeds [39]. RY elements confer seed-specific expression in *Arabidopsis*, legumes, and tobacco. Deletion or mutation of RY elements drastically reduces seed-specific promoter activity [42]. Speculatively, the CARE and RY elements included in *SlPRX* gene promoters indicate the potential roles of *SlPRXs* in tomato seed development and germination.

### 3.6. PRXs Are Involved in Tomato Seed Germination

**Figure 8:**
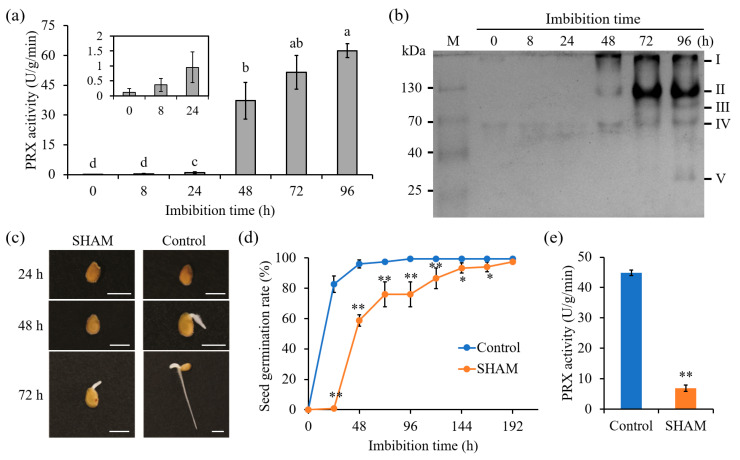
SlPRXs are involved in tomato seed germination. (**a**) Dynamic changes in PRX enzyme activity during tomato seed germination. Different lowercase letters in the graphs indicate significant differences between different time points as determined by one-way ANOVA (*p* < 0.05). (**b**) The detection of PRX isozymes in germinating tomato seeds. The Roman numerals I–V represent the five PRX isozymes. (**c**,**d**) The effects of SHAM on tomato seed germination phenotypes (**c**) and germination rate. Scale bars in (**c**) indicate 5 mm. (**e**) PRX activity was drastically inhibited in SHAM-treated seeds. The data in (**d**,**e**) are presented as the mean ± SD, and asterisks indicate significant differences compared with the control according to Student’s *t*-test (* *p* < 0.05, ** *p* < 0.01).

Previous studies have demonstrated that five *AtPRXs* play important roles in early seed germination in *Arabidopsis*, with *AtPRX16*, *AtPRX62*, *AtPRX69*, and *AtPRX71* having a repressive role on testa rupture and micropylar endosperm rupture, and *AtPRX4* having the opposite activating role on both processes. [2]. To verify the roles of *SlPRXs* in tomato seed germination, the changes in PRX enzymatic activities were examined. Figure 8a shows that during 24 h of tomato seed germination, only trace activities of PRX were observed. PRX activity increased substantially at 48 h and then gradually increased until 96 h. Additionally, non-deformable PAGE electrophoresis was utilized for analysis (Figure 8b). Obvious bands were detected until 48 h after germination, consistent with the enzymatic activity assay. In total, five isozymes were identified, named I, II, III, IV, and V according to their molecular weight. Among these isozymes, I, II, and IV were detected as early as 48 h and gradually increased thereafter. Isozyme III was found at 72 h, and isozyme V was observed only at 96 h (Figure 8b).

Furthermore, SHAM, a PRX enzyme inhibitor [25], was applied to tomato seeds. Treatment with SHAM drastically inhibited seed germination (Figure 8c,d) and repressed PRX activity (Figure 8e). These results suggest that PRXs are significantly involved in tomato seed germination.

### 3.7. Dynamic Expression Patterns of SlPRX Genes Indicate Stage-Specific Functional Modules During Seed Germination

**Figure 9:**
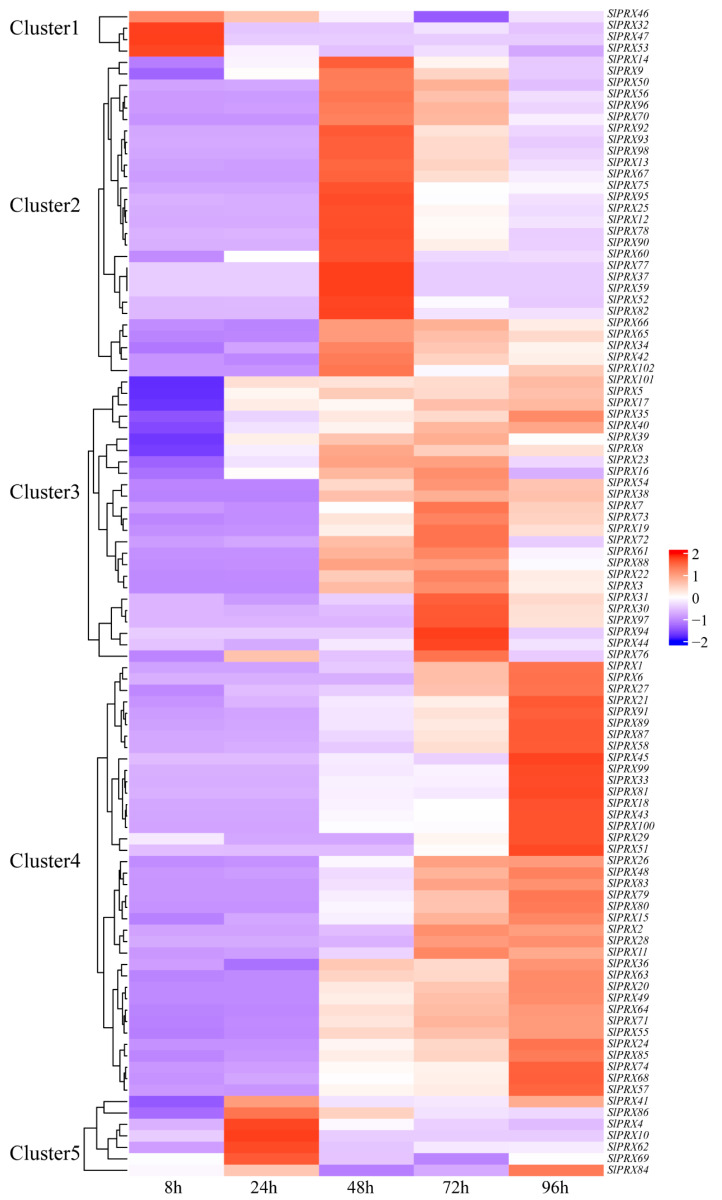
The expression patterns of 102 *SlPRX* genes during tomato seed germination. A heatmap of the expression patterns of *SlPRX* genes across different germination stages, based on FPKM values from RNA-seq data. The heatmap was visualized by using R studio. The expression levels are represented by a purple-to-red color scheme, with a redder color denoting a higher level of expression. The FPKM values for each *SlPRX* gene are available in Supplementary Table S4.

**Figure 10:**
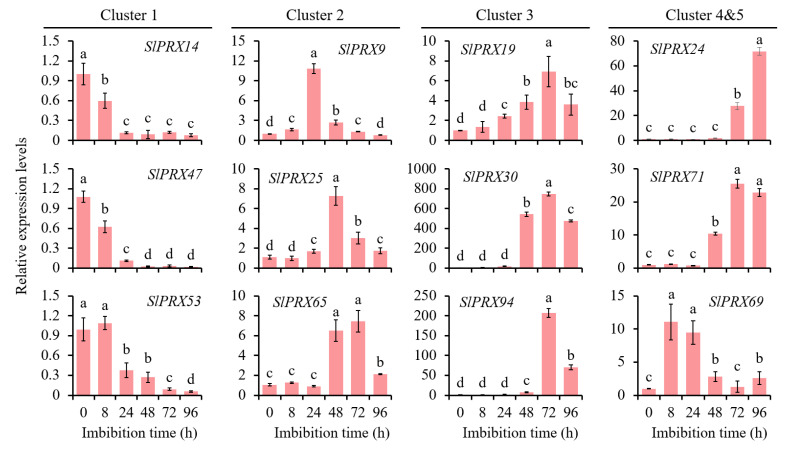
qRT-PCR validation of the expression of selected *SlPRX* genes during tomato seed germination. The expression level at 0 h was set to 1.0 for each gene. Different lowercase letters for each gene indicate statistically significant changes in expression levels across the time courses (*p* < 0.05).

To characterize the functions of *SlPRX* members in tomato seed germination, RNA-seq analysis was carried out at different germination time points (8, 24, 48, 72, and 96 h). Figure 9 shows that all the 102 *SlPRX* genes were expressed during tomato seed germination, but with different patterns. The 102 *SlPRX* genes were classified into five clusters according to their expression patterns. Cluster 1 contained four genes, *SlPRX32*, *SlPRX46*, *SlPRX47*, and *SlPRX53*, which were only highly expressed as early as 8 h after seed germination. A total of 28 *SlPRX* genes were classified as Cluster 2, whose expression levels peaked at 48 h of seed germination. Cluster 3 contained 25 *SlPRX* genes. The expression levels of Cluster 3 genes increased at 48 h, peaked at 72 h, and then declined. A total of 38 *SlPRX* genes were classified as Cluster 4, which is the largest cluster. The genes in Cluster 4 increased at 24 or 48 h of seed germination, and remained elevated for the following 48 h. In Cluster 5, seven *SlPRX* genes were included. Of them, five genes (*SlPRX4*, *SlPRX10*, *SlPRX62*, *SlPRX69*, and *SlPRX86*) were highly expressed at only 24 h, and two genes (*SlPRX41* and *SlPRX84*) showed high expression levels at 24 and 96 h (Figure 9). Moreover, the expression dynamics of nine *SlPRX* genes from different clusters were validated by qRT-PCR. These genes displayed expression patterns consistent with the results of transcriptome analysis (Figure 10). Taken together, these results indicate that tomato seed germination is finely controlled by coordination and relay between multiple *SlPRX* genes.

## 4. Discussion

Class III peroxidases (PRXs) are widely involved in plant growth, development, and responses to both biotic and abiotic stresses [14,16]. Although comprehensive deciphering of the *PRX* gene family has been reported in several plant species, including Arabidopsis, rice, and maize, comprehensive characterization of this gene family in tomato has not been reported. In this study, we performed a genome-wide identification and analysis of the *PRX* gene family in tomato. A total of 102 *SlPRX* genes were identified. This number is comparable with that reported in tobacco (210) [8], rice (138) [16], soybean (124) [8], maize (119) [10], and potato (102) [11], but higher than that in pear (94) [43], sugarcane (82) [44], pepper (75) [37], carrot (75) [17], *Arabidopsis* (73) [45], *Populus simonii* (69) [46], and grape (47) [47]. Conversely, it is much lower than the PRX gene count in wheat (374) [14]. The significant differences in *PRX* gene family size between various plant species are likely due to genome duplications and environmental adaptive pressures. After duplication, genes generally tend to pseudogenize or undergo neofunctionalization to break down functional redundancy [48,49]. It is plausible that some of the 102 members identified in this study may represent pseudogenes or non-functional duplicates. Future functional studies, such as transcriptomic validation or assessment of evolutionary constraints, would help clarify the functional status of these genes and refine the true repertoire of active *SlPRX* genes.

The chromosomal localization analysis indicated the uneven distribution of the *SlPRX* gene family members across chromosomes, with a higher number of *SlPRX* genes found on chromosome 2, while fewer *SlPRX* genes were detected on chromosomes 6, 8, and 9 (Figure 1). The concentration of genes on chromosome 2 may indicate a historical duplication event, such as segmental or whole-genome duplication, leading to the expansion of this gene family. In contrast, the scarcity of members on chromosomes 6, 8, and 9 could reflect selective constraints, lower rates of duplication, or gene loss events in this region. Additionally, we found the preferential localization of genes at the end of each chromosome (Figure 1). This might be linked with the features of subtelomeric regions, which are known for their high recombination rates and epigenetic variability [50]. This distribution pattern could facilitate gene diversification, regulatory flexibility, or adaptive evolution.

Further collinearity analysis elucidated the mechanisms driving the biased genomic arrangement. The findings revealed that tomato shared 99 pairs of homologous genes with potato, was an increase of 47 pairs compared with its homologous genes with *Arabidopsis* (Figure 6). This finding aligns with the closer evolutionary relationship between tomato and potato, as both belong to the *Solanum* genus. The phylogenetic tree analysis revealed that the *SlPRX* gene family is divided into five groups, with genes within each group sharing similar exon–intron structures (Figure 2 and Figure 3), suggesting functional conservation or divergence between these groups. Furthermore, all SlPRX members contain the typical peroxidase domain, and the majority of these SlPRX members possess all ten motifs (94/102), suggesting a common functional core between these proteins related to their peroxidase activity. The diverse protein motifs likely determine their involvement in different regulatory pathways and the execution of distinct biological functions [51].

*Cis*-regulatory elements are crucial in gene expression regulation by acting as binding sites for transcription factors. In this study, we identified a variety of *cis*-regulatory elements in the promoter regions of *SlPRX* genes, including motifs responsive to light, hormones, defense, and stress. The presence of light- and hormone-responsive elements such as G-box, TGA-element, and ABRE is consistent with canonical regulatory motifs previously characterized in *PRX* genes from *Arabidopsis* [45], rice [16], and *Populus simonii* [46], indicating conserved transcriptional control mechanisms in plant development and responses to abiotic stimuli. Importantly, we also observed a notable distribution of CARE and RY elements in the promoter regions of *SlPRX* genes. To our knowledge, CARE is the regulatory element for GA-inducible expression of hydrolase genes in germinating seeds [39]. RY elements have not been reported in the regulation of gene expression during late embryogenesis and seed development via ABI3 and FUS3 transcription factors [41]. This suggests functional adaptation of SlPRXs associated with the regulation of seed germination.

**Figure 11:**
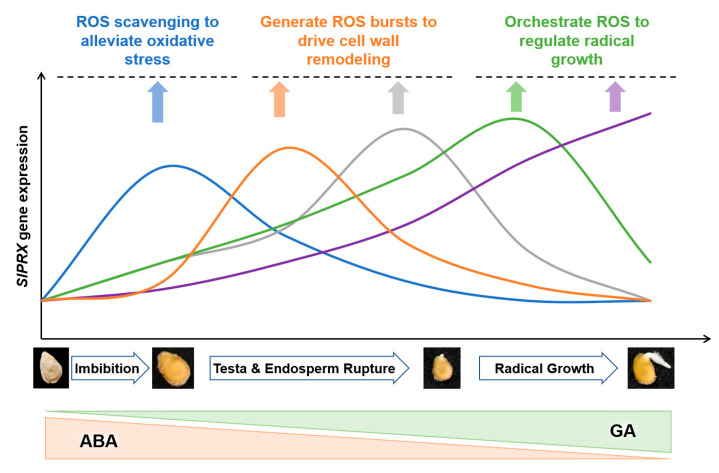
A schematic model of the proposed SlPRX–ROS–hormone interplay during tomato seed germination. The temporal expression of five *SlPRX* gene clusters across three key germination phases is depicted, highlighting how PRXs fine-tune the oxidative window to regulate processes from imbibition to radicle growth.

The present study has also demonstrated that *SlPRX* genes exhibit stage-specific induction patterns during seed germination, with distinct expression peaks at 0–96 h post imbibition (Figure 9 and Figure 10). This temporal expression pattern was consistent with the suppression of germination under PRX inhibitor treatment and the concurrent rise in enzymatic activity (Figure 8). These results strongly implicate the critical roles of PRXs in seed germination. The phased induction of these genes suggests functional diversification within this gene family, with each cluster fine-tuning ROS homeostasis at distinct developmental stages. Notably, SlPRXs likely mediate their effects through spatially and temporally controlled reactive oxygen species (ROS) signaling (Figure 11). During early germination (0–8 h), their activity might promote ROS scavenging to alleviate oxidative stress during imbibition, while later peaks (24–48 h) could generate localized ROS bursts to drive cell wall remodeling via cross-link cleavage or lignin modification. Then, the stage between 72 and 96 h might orchestrate ROS signaling to regulate radical elongation. It has been known that ROS can function as both a toxic byproduct and a signaling molecule [52]. Recent studies have further highlighted ROS as an integrative signaling hub in seed germination. ROS can interact with ethylene, abscisic acid (ABA), and gibberellin (GA) homeostasis, as well as affecting Ca^2+^ signaling, NO signaling, and the MAPK cascade [21,53]. Moreover, ROS can function as a regulator in nuclear genome remodeling and as epigenetic modifiers [54]. Optimal germination requires tight control of oxidative thresholds. Consistent with this, we found that a PRX inhibitor could arrest seed germination. One possible explanation is that disrupted peroxidase activity impairs ROS balance, either causing oxidative damage or suppressing ROS-dependent signaling cascades [55,56].

Furthermore, the sequential expression of *SlPRX* genes points to their specialized roles in crosstalk with other germination triggers. Early-induced *SlPRX* genes might interact with ABA and GA hormone pathways, while late-phase isoforms could facilitate radicle emergence by weakening micropylar endosperm tissues [2]. The interaction of SlPRXs and the ABA/GA ratio during seed germination may include a dual mechanism. On one hand, SlPRXs potentially fine-tune the ABA/GA balance, which is central to dormancy release and germination initiation. The early clusters repress ABA signaling or enhance GA sensitivity, and the late-stage genes reinforce cell wall loosening in synergy with GA-mediated hydrolases. Consistent with this, a study on primed tomato seeds revealed that H_2_O_2_ enhances seed germination capacity by reducing the ABA/GA_3_ ratio through upregulation of the expression of the GA biosynthesis gene *GA3ox1* and the ABA catabolism gene ABA 8-hydroxylase [57]. On the other hand, the expression of these *SlPRX*s may be regulated by the spatiotemporal distribution of ABA and GA in tomato seeds. However, these above speculations still need further validation.

From a practical perspective, understanding the roles of specific SlPRX clusters in ABA/GA crosstalk and ROS management would offer a promising avenue for breeding and biotechnology. Manipulating *SlPRX* expression could enhance seed vigor, synchronize germination under stress conditions, or improve seedling establishment. Selecting for these candidate *SlPRX* genes might help develop new tomato varieties with improved germination performance or stress resilience, contributing to more sustainable tomato production.

## 5. Conclusions

In this study, a total of 102 class III peroxidase (*SlPRX*) genes were identified in the tomato genome through genome-wide analysis. These genes are unevenly distributed across all 12 chromosomes. Based on phylogenetic relationships, the *SlPRX* genes were classified into five distinct groups. The members of each group exhibited similar exon–intron organization and motif arrangements. The intraspecific and interspecific collinearity analysis indicated that multiple duplication events of the *SlPRX* family were the main driving force leading to its expansion. Promoter analysis revealed the presence of multiple *cis*-acting elements related to abiotic and biotic stress responsiveness, phytohormone signaling, and growth and development. Expression profiling during seed germination demonstrated that *SlPRX* genes exhibit diverse expression patterns, and according to their expression peaks, the 102 *SlPRX* genes could be classified into five clusters. Furthermore, the involvement of PRXs in the regulation of seed germination was confirmed by the enzymatic activity assay and the inhibitory effects of SHAM (the inhibitor of PRXs) on seed germination. Notably, many previous studies have strongly associated PRXs with stress responses, which highlights their potential application in improving germination resilience. These candidate *SlPRX* genes could serve as promising targets for genetic engineering or marker-assisted breeding with the aim of developing new tomato cultivars with enhanced germination performance. Overall, this study presents a comprehensive investigation of the *SlPRX* gene family in tomato and lays a solid foundation for further studies on its biological roles in seed germination.

## Data Availability

The original contributions presented in this study are included in the article/Supplementary Material. Further inquiries can be directed to the corresponding author.

## Supplementary Materials

The following supporting information can be downloaded at: https://www.mdpi.com/article/10.3390/antiox14111310/s1, Table S1: List of primers for qRT-PCR in this study. Table S2: List of all class III peroxidase genes identified in tomato genome. Table S3: Information of putative cis-elements in promoter regions of SlPRX gene family. Table S4: The FPKM values of SlPRX genes during tomato seed germination. Figure S1: The sequence logos of 10 conserved motifs in SlPRX proteins were analyzed with the MEME program (http://meme-suite.org/ accessed on 8 November 2024). Figure S2: Number of categories of *cis*-acting elements among *SlPRX* genes.

## Abbreviations

The following abbreviations are used in this manuscript:

PRX	Class III peroxidase
ROS	Reactive oxygen species
SHAM	Salicylhydroxamic acid
PAGE	Polyacrylamide gel electrophoresis
FPKM	Kilobase fragment count per million mapped reads per transcript
p*I*	Isoelectric point
Mw	Molecular weight
LTR	Low temperature-responsive element
DRE	Drought and high-salinity stress
ABRE	Abscisic acid-responsive element
GARE	Gibberellin-responsive element
