# Supplementary material for: Deciphering the Class III Peroxidase Gene Family and Verifying Their Expression in Modulating Seed Germination in Tomato
Source: Antioxidants (Basel). 2025 Oct 30;14(11):1310. doi: 10.3390/antiox14111310 (PMC12649747; doi:10.3390/antiox14111310)
Supplement: Supplementary file 1 [file antioxidants-14-01310-s001.zip › revised 2025 Antioxidants-Supplementary Figures S1-S2.pdf]

Supplementary Figures

Figure S1.

	E-value	Sites	Width
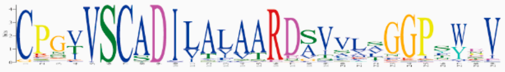	1.6e-1894	101	29
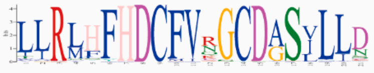	3.7e-1625	97	21
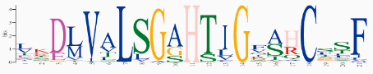	3.5e-1205	101	21
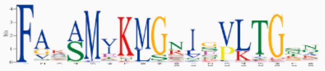	5.1e-752	90	18
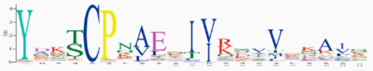	8.7e-702	95	21
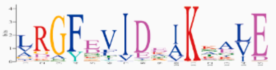	4.4e-639	102	15
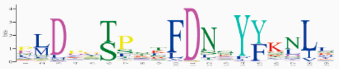	2.5e-651	102	19
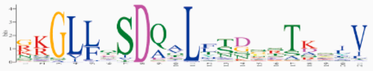	8.1e-708	101	21
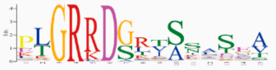	8.6e-581	102	15
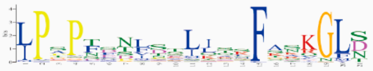	6.1e-650	101	21

Figure S1. The sequence logos of 10 conserved motifs in SlPRX proteins were analyzed with the MEME program (http://meme-suite.org/). Each letter represents an amino acid, and the height of letters in the *y*-axis indicate the degree of conservation of the amino acid. The *x*-axis represents the number of amino acids. Motif 1, motif 6 and motif 8 are MYB domains.

Figure S2.

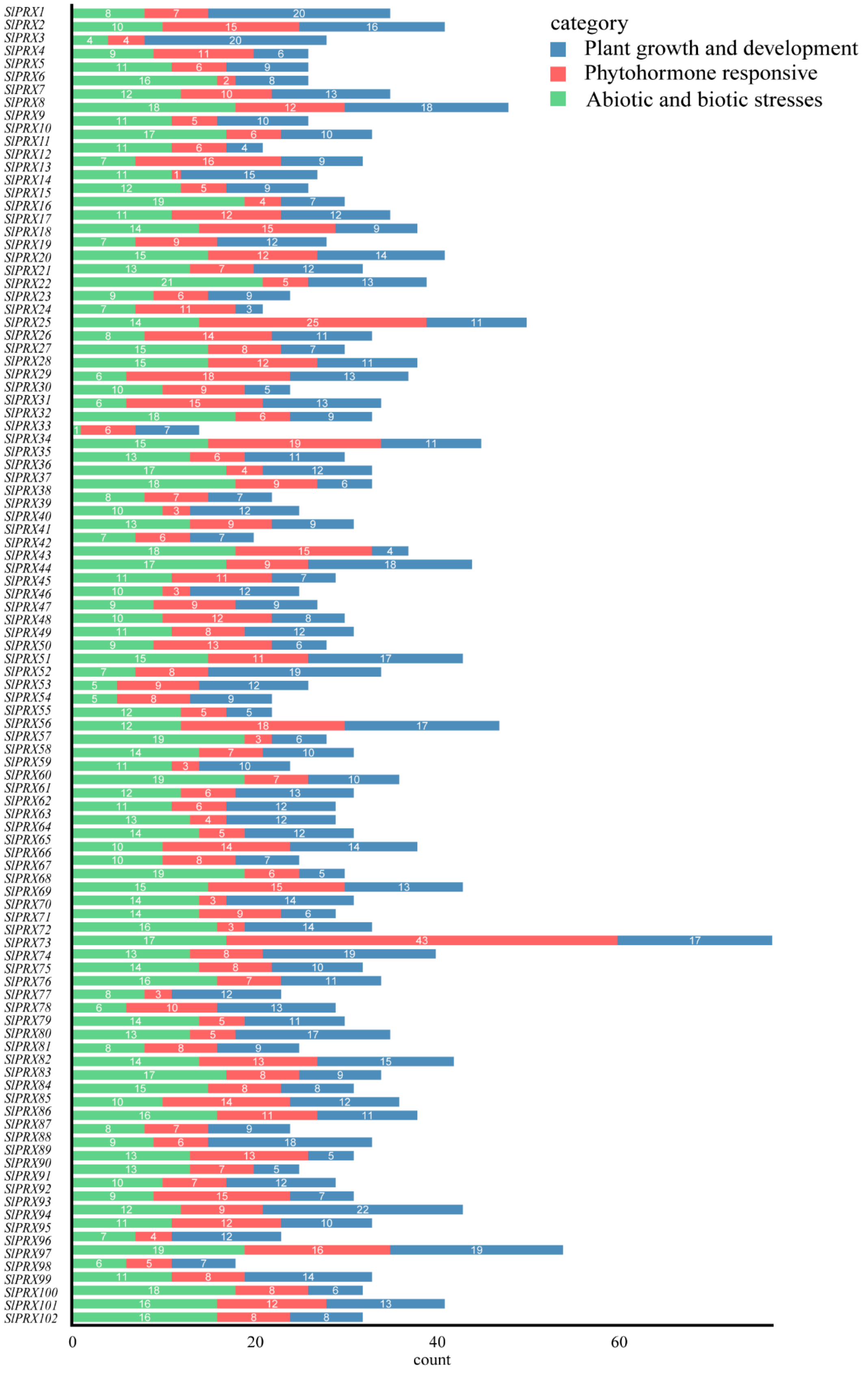

Figure S2. Number of categories of cis-acting elements among *SlPRX* genes. The different colored histogram represents the sum of the cis-acting elements in each category.
